# Associations of the adrenomedullin gene polymorphism with prehypertension and hypertension in Lithuanian children and adolescents: a cross-sectional study

**DOI:** 10.1038/s41598-019-43287-3

**Published:** 2019-05-02

**Authors:** Sandrita Simonyte, Renata Kuciene, Virginija Dulskiene, Vaiva Lesauskaite

**Affiliations:** Institute of Cardiology of the Medical Academy, Lithuanian University of Health Sciences, Sukileliu 15, LT-50161 Kaunas, Lithuania

**Keywords:** Genotype, Epidemiology

## Abstract

The aim of this study was to evaluate the association of *ADM* genetic variant and HBP among Lithuanian adolescents aged 12–15 years. This is a cross-sectional study of a randomly selected sample of 675 12–15-years-old schoolchildren who were surveyed during November 2010 to April 2012 in the baseline survey. Single-nucleotide polymorphism (SNP) of *ADM* gene (rs7129220) was evaluated using real-time PCR. Logistic regression analyses were used to test the associations of *ADM* (rs7129220) polymorphism with HBP under four inheritance models based on the Akaike Information Criterion (AIC) and to calculate the odds ratios. In the multivariate analysis, boys carrying *ADM* AG genotype (vs. carriers of *ADM* GG genotype), *ADM* AG + AA genotype (vs. carriers of *ADM* GG genotype) and *ADM* AG genotype (vs. carriers of *ADM* GG + AA genotype) had higher odds of having hypertension in codominant, dominant, and overdominant inheritance models. Girls with *ADM* AG + AA had increased odds of prehypertension compared to girls with the *ADM* GG genotype carriers in dominant inheritance model. Significant associations were observed in additive models separately for boys (hypertension) and girls (prehypertension). Our results indicate that *ADM* gene polymorphism was significantly associated with higher odds of HBP in Lithuanian adolescents aged 12–15 years.

## Introduction

High blood pressure affects almost a half of the adult population worldwide and is one of the major risk factors of cardiovascular disease^1^. Even though, according to various studies, the prevalence of hypertension among children is increasing^2,3^ and in some cases reaches 30%^4^, the prevalence of hypertension in preadolescents and adolescents has slightly decreased during the last 2 decades^5^.

The prevalence of overweight and obesity is increasing in economically developed countries. This tendency is observed not only in adults but also in children and adolescents^6^. Elevated BP with overweight and obesity in childhood are related to the risk of adult hypertension^7^ and the risk of cardiovascular morbidity and mortality^8^. More than 70% of obese teenagers are continuing to be obese in adulthood. European Youth Heart Study indicated that males have a higher risk of obesity in adolescence^9^.

Despite the fact that lifestyle modification could prevent hypertension, almost 40–60% of individual differences in BP have a genetic basis^10,11^. In addition to environmental factors, a series of genome-wide association studies (GWAS) have identified new loci and genes linked with the risk of elevated resting BP and hypertension in various populations^12–17^. However, it remains unclear how these genetic variation influence blood pressure.

In this study SNP rs7129220 of adrenomedullin (*ADM)* gene was chosen according to the previously reported data from GWAS studies^14–16^. These studies have shown that SNP rs7129220 of ADM has association with blood pressure^14^, systolic^15^ and diastolic blood pressure^16^. Epidemiological studies are providing data on the high prevalence of increased BP or hypertension in children (21%) and adolescents (35%) in Lithuania^18,19^. Thus, the study is aimed to replicate data obtained in GWAS in children and adolescents from the population with highly prevalent hypertension.

Adrenomedullin (*ADM)* plays a critical role in blood pressure homeostasis and has a wide range of biological functions, including vasodilatation, natriuresis, and stimulation of nitric oxide (NO)^20–22^. *ADM*, a potent vasodilator peptide, was first discovered in 1993 in human tissue samples of adrenal glands affected by pheochromocytoma, and as a circulating plasma hormone is expressed in tissues relevant to cardiovascular and renal function, including blood vessels, heart, kidneys, brain, and lung^23,24^. Data from studies have shown that plasma ADM level increases in patients with cardiovascular diseases, including hypertension, heart failure, and chronic renal failure^25–27^. ADM can reduce total peripheral resistance, leading to decreased blood pressure and increased heart rate^28^. Its role in blood pressure regulation has been examined in numerous studies^20,22,29^, which demonstrated that adrenomedullin could be a promising biomarker for cardiovascular disease in the future^30–32^.

## Results

The demographic, anthropometric, and BP characteristics data of 675 adolescents (332 boys and 343 girls) are shown in Table 1. The mean ± SD age of all study subjects was 13.27 ± 1.14 years and did not differ between boys and girls (Table 1). Mean values of weight, height, WC, SBP, and PP were significantly higher in boys than in girls. The girls had significantly higher mean DBP levels than boys did. No significant difference was found between these two groups in mean BMI or in mean MAP. The frequency of *ADM* genotypes and alleles between boys and girls did not differ significantly either.

**Table 1:** Demographic, anthropometric, and BP characteristics, and the frequency (%) of *ADM* genotypes and alleles of the study subjects by sex.

Variables	Total (n = 675)	Boys (n = 332)	Girls (n = 343)	P*
Age, years, mean (SD)	13.27 (1.14)	13.31 (1.15)	13.22 (1.13)	0.433
Weight, kg, mean (SD)	52.56 (12.18)	54.14 (13.23)	51.03 (10.87)	0.001
Height, cm, mean (SD)	162.92 (9.48)	164.47 (10.56)	161.42 (8.04)	<0.001
BMI, kg/m^2^, mean (SD)	19.62 (3.35)	19.80 (3.46)	19.44 (3.24)	0.261
WC, cm, mean (SD)	67.41 (8.61)	69.59 (8.82)	65.30 (7.84)	<0.001
SBP, mmHg, mean (SD)	120.11 (14.68)	123.18 (16.03)	117.13 (12.57)	<0.001
DBP, mmHg, mean (SD)	64.94 (8.00)	64.20 (7.97)	65.66 (7.97)	0.017
MAP, mm Hg, mean (SD)	83.31 (8.98)	83.84 (9.28)	82.80 (8.66)	0.08
PP, mm Hg, mean (SD)	55.16 (12.34)	58.98 (13.72)	51.47 (9.48)	<0.001
**Age, years, n (%)**				
12–13	403 (59.7)	196 (59.0)	207 (60.3)	0.754
14–15	272 (40.3)	136 (41.0)	136 (39.7)	
**BMI categories, n (%)**				
Normal weight	566 (83.9)	275 (82.8)	291 (84.8)	0.530
Overweight/obesity	109 (16.1)	57 (17.2)	52 (15.2)	
**WC percentile categories, n (%)**				
<75^th^	587 (87.0)	284 (85.5)	303 (88.3)	0.304
≥75^th^	88 (13.0)	48 (14.5)	40 (11.7)	
***ADM*** **genotypes, n (%)**				
GG	544 (80.6)	275 (82.8)	269 (78.4)	0.099
AG	124 (18.4)	56 (16.9)	68 (19.8)	
AA	7 (1.0)	1 (0.3)	6 (1.8)	
***ADM*** **allele frequency (%)**				
G	89.8	91.3	88.3	0.076
A	10.2	8.7	11.7	

*Boys *vs*. girls (significant differences between the groups were found by applying the t-test for continuous variables and the chi-square (χ^2^) test for categorical variables).

*Abbreviations: ADM*, adrenomedullin; BP, blood pressure; BMI, body mass index; WC, waist circumference; SBP, systolic blood pressure; DBP, diastolic blood pressure; MAP, mean arterial pressure; PP, pulse pressure; SD, standard deviation.

The prevalence of prehypertension and hypertension was 5.9% and 31.0% (6.3% and 38.9% among boys; 5.5% and 23.3% among girls), respectively. Girls were more likely to have NBP than boys (71.2% vs. 54.8%; P < 0.001). Adolescents aged 14–15 years were more likely to have HBP than adolescents aged 12–13 years (39.3% vs. 35.3%). However, statistically significant differences were observed only among boys (54.5% vs. 38.8%, P < 0.05), (data not shown). The prevalence of prehypertension and hypertension was higher among overweight/obese subjects and subjects with high WC (≥75^th^ percentile) compared to those with normal BMI and WC (<75^th^ percentile) (p < 0.001). Prehypertensive and hypertensive participants had significantly higher mean values for weight, BMI, and WC compared to normotensive participants. The mean value for height was significantly higher among hypertensive subjects compared with subjects with NBP. There was no significant difference in mean value of age between these groups (Table 2). Carriers of the A allele were more common among children with prehypertension and hypertension in comparison to the NBP group. Significant differences in the frequencies of the *ADM* alleles were observed in boys: NBP vs. hypertension (P = 0.027), and in girls: NBP vs. prehypertension (P = 0.016) (data not shown).

**Table 2:** Characteristics of the study participants according to BP level.

Variables	NBP	Prehypertension	Hypertension	P value
Age, years, mean (SD)	13.26 (1.15)	13.30 (1.31)	13.29 (1.09)	0.825
BMI, kg/m^2^, mean (SD)	18.66 (2.83)	20.34 (2.84)^a^	21.43 (3.66)^a^	<0.001
WC, cm, mean (SD)	64.60 (6.92)	70.80 (6.89)^a^	72.48 (9.44)^a^	<0.001
Weight, kg, mean (SD)	49.24 (10.82)	55.06 (12.13)^a^	58.86 (12.26)^a^	<0.001
Height, cm, mean (SD)	161.67 (9.66)	163.73 (11.18)	165.31 (8.26)^a^	<0.001
SBP, mm Hg, mean (SD)	110.89 (7.34)	124.39 (4.14)^a^	138.07 (9.14)^a,b^	<0.001
DBP, mm Hg, mean (SD)	62.23 (6.62)	67.33 (7.20)^a^	70.01 (8.14)^a^	<0.001
MAP, mm Hg, mean (SD)	78.43 (5.89)	86.33 (5.09)^a^	92.67 (6.86)^a,b^	<0.001
PP, mm Hg, mean (SD)	48.66 (7.49)	57.06 (8.06)^a^	68.06 (10.58)^a,b^	<0.001
**Sex, n (%)**				
Boys	182 (42.7)	21 (52.5)	129 (61.7)*	<0.001
Girls	244 (57.3)	19 (47.5)	80 (38.3)*	
**Age, years, n (%)**				
12–13	261 (61.3)	22 (55.0)	120 (57.4)	0.534
14–15	165 (38.7)	18 (45.0)	89 (42.6)	
**BMI categories, n (%)**				
Normal weight	387 (90.8)	28 (70.0)*	151 (72.2)*	<0.001
Overweight/Obesity	39 (9.2)	12 (30.0)*	58 (27.8)*	
**WC percentile categories, n (%)**				
<75^th^	402 (94.4)	32 (80.0)*	153 (73.2)*	<0.001
≥75^th^	24 (5.6)	8 (20.0)*	56 (26.8)*	
***ADM*** **genotypes, n (%)**				
GG	352 (82.6)	30 (75.0)	162 (77.5)	0.395
AG	71 (16.7)	9 (22.5)	44 (21.1)	
AA	3 (0.7)	1 (2.5)	3 (1.4)	
***ADM*** **allele frequency (%)**				
G	91.0	86.3	88.0	0.15
A	9.0	13.7	12.0	

Means were compared using the t-test and ANOVA.

The chi-square (χ^2^) test was used for categorical variables.

^a^P < 0.05 *vs*. the NBP group.

^b^P < 0.05 *vs*. the prehypertension group.

*P < 0.05 *vs*. the NBP group (z test).

*Abbreviations: ADM*, adrenomedullin; SBP, systolic blood pressure; DBP, diastolic blood pressure; MAP, mean arterial pressure; PP, pulse pressure; NBP, normal blood pressure; BMI, body mass index; WC, waist circumference; SD, standard deviation.

The characteristics of study subjects according to the *ADM* genotypes are presented in Table 3. Mean values of age, weight, height, BMI, and WC were similar in different genotype carrier groups. Carriers of the AG genotype had significantly higher SBP, DBP, and MAP in comparison to wild-type homozygous study subjects. The highest values of SBP, DBP, and MAP were observed in the AA genotype carriers in comparison to AG and GG genotype carriers.

**Table 3:** Characteristics of the study population (means and SD) according to *ADM* genotypes.

Characteristics	*ADM* genotypes			P value (ANOVA)
	GG (n = 544)	AG (n = 124)	AA (n = 7)	
Age, years, mean (SD)	13.25 (1.13)	13.32 (1.21)	13.57 (0.98)	0.496
Weight, kg, mean (SD)	52.45 (12.27)	52.70 (11.99)	58.57 (6.95)	0.210
Height, cm, mean (SD)	162.81 (9.66)	163.08 (8.78)	169.07 (4.87)	0.216
BMI, kg/m^2^, mean (SD)	19.60 (3.36)	19.65 (3.40)	20.46 (1.96)	0.452
WC, cm, mean (SD)	67.48 (8.76)	67.10 (8.02)	67.50 (7.17)	0.903
SBP, mm Hg, mean (SD)	119.58 (14.64)	122.20 (14.92)^a^	123.86 (10.42)	0.091
DBP, mm Hg, mean (SD)	64.46 (8.01)	66.65 (7.58)^a^	72.00 (8.01)^a,b^	0.001
MAP, mm Hg, mean (SD)	82.82 (8.97)	85.15 (8.80)^a^	89.27 (8.06)^a^	0.007
PP, mm Hg, mean (SD)	55.12 (12.34)	55.55 (12.56)	51.86 (7.94)	0.840

^a^P < 0.05 *vs*. the *ADM* GG genotype (t-test).

^b^P < 0.05 vs. the *ADM AG genotype* (*t*-*test*).

*Abbreviations: ADM*, adrenomedullin; BMI, body mass index; WC, waist circumference; SBP, systolic blood pressure; DBP, diastolic blood pressure; MAP, mean arterial pressure, PP, pulse pressure; SD, standard deviation.

The female carriers of the minor allele (AG + AA) with NBP had a significantly higher SBP than wild-type homozygous genotype carriers did.

There were no significant differences in allele and genotype frequencies between the HBP and NBP groups in the Hardy-Weinberg equilibrium (P > 0.05).

Finally, we analysed associations between high blood pressure and the *ADM* polymorphism by inheritance models (Tables 4 and 5). According to the univariate logistic regression, the additive model fitted best for the association of *ADM* rs7129220 with prehypertension in girls (OR 2.58, CI 95% 1.15–5.81) and hypertension in boys (OR 1.93, CI 95% 1.09–3.43). Significant associations were also found in the dominant model in girls with prehypertension and in the codominant, dominant, and overdominant models in boys with hypertension (Table 4). Finally, we controlled potential confounding variables by calculating aORs adjusted by BMI and WC (Table 5). The value of significant crude OR tended to increase slightly after adjusting by BMI and WC.

**Table 4:** Associations between the *ADM* gene polymorphism and high blood pressure by sex (univariate analyses).

Variables	*ADM* genotypes	Prehypertension	P value	AIC	Hypertension	P value	AIC
Models		OR (95% CI)			OR (95% CI)		
***Boys***							
Codominant	GG	1.00			1.00		
	AG	0.66 (0.15–3.01)	0.593	138.72	**1.84 (1.02–3.32)**	**0.043**	422.20
	AA						
Dominant	GG	1.00			1.00		
	AG + AA	0.66 (0.15–3.01)	0.593	138.72	**1.90 (1.06–3.42)**	**0.032**	421.43
Overdominant	GG + AA	1.00			1.00		
	AG	0.66 (0.15–3.01)	0.593	138.72	**1.82 (1.01–3.29)**	**0.047**	422.09
Additive		0.66 (0.15–3.01)	0.593	138.72	**1.93 (1.09–3.43)**	**0.024**	420.94
***Girls***							
Codominant	GG	1.00			1.00		
	AG	2.70 (0.99–7.34)	0.052	137.73	1.01 (0.53–1.93)	0.978	367.60
	AA	5.91 (0.57–61.54)	0.137		2.06 (0.34–12.63)	0.433	
Dominant	GG	**1.00**			1.00		
	AG + AA	**2.89 (1.11–7.58)**	**0.031**	136.00	1.07 (0.58–2.00)	0.822	366.13
Overdominant	GG + AA	1.00			1.00		
	AG	2.51 (0.94–6.73)	0.067	137.37	0.99 (0.52–1.90)	0.984	366.18
Additive		**2.58 (1.15–5.81)**	**0.022**	135.75	1.13 (0.65–1.95)	0.674	366.01

*Abbreviations: ADM*, adrenomedullin; OR, odds ratio; CI, confidence interval.

**Table 5:** Multivariate logistic regression analysis of *ADM* gene polymorphism and prehypertension and hypertension by sex.

Variables	*ADM* genotypes	Prehypertension	P value	AIC	Hypertension	P value	AIC
Models		aOR* (95% CI)			aOR* (95% CI)		
***Boys***							
Codominant	GG	1.00			1.00		
	AG	0.61 (0.13–2.83)	0.525	139.96	**2.00 (1.08–3.68)**	**0.027**	399.82
	AA						
Dominant	GG	1.00			1.00		
	AG + AA	0.61 (0.13–2.83)	0.525	139.60	**2.08 (1.13–3.81)**	**0.019**	399.23
Overdominant	GG + AA	1.00			1.00		
	AG	0.61 (0.13–2.83)	0.525	139.96	**1.97 (1.07–3.63)**	**0.030**	340.06
Additive		0.61 (0.13–2.83)	0.525	139.96	**2.11 (1.17–3.82)**	**0.014**	398.63
***Girls***							
Codominant	GG	1.00			1.00		
	AG	2.64 (0.91–7.73)	0.076	129.72	1.03 (0.52–2.05)	0.925	338.45
	AA	8.95 (0.83–96.31)	0.071		2.99 (0.49–18.51)	0.237	
Dominant	GG	**1.00**			1.00		
	AG + AA	**2.95 (1.06–8.25)**	**0.039**	128.53	1.14 (0.59–2.19)	0.701	337.56
Overdominant	GG + AA	1.00			1.00		
	AG	2.42 (0.85–6.92)	0.099	129.99	1.01 (0.51–1.99)	0.983	347.70
Additive		**2.79 (1.17–6.64)**	**0.021**	127.75	1.22 (0.68–2.18)	0.501	337.26

aOR*, adjusted odds ratio for the body mass index and waist circumference.

*Abbreviations: ADM*, adrenomedullin; AIC, Akaike information criterion; aOR, adjusted odds ratio; CI, confidence interval.

## Discussion

The present study investigated the associations of the *ADM* gene polymorphism (rs7129220) with high blood pressure among Lithuanian adolescents aged 12–15 years. In Lithuania, the Kaunas Cardiovascular Risk Cohort study (35-year follow-up) examined the relationships between the genetic risk score of four single nucleotide polymorphisms (including *ADM*) and hypertension among middle-aged adults^33^.

According to our data, the prevalence rates of prehypertension and hypertension among Lithuanian schoolchildren were 5.9% and 31.0%, respectively. Our results are partially consistent with those of other studies conducted on various age groups and in different populations of children and adolescents. These studies also demonstrated a high prevalence of HBP – for instance, in Lithuanian children and adolescents aged 12–15 years (the prevalence of prehypertension was 12.8% and the prevalence of hypertension – 22.2%)^19^, in Greek schoolchildren aged 9–13 years (prehypertension – 14.2%; hypertension – 23%)^34^, in Portuguese children and adolescents aged 4–18 years (high-normal BP – 21.6% and stage 1 hypertension – 12.8%)^35^, in Chinese children and adolescents aged 5–18 years (prehypertension – 15.2% and hypertension – 20.5%)^36^, in Spanish children aged 4–6 years (prehypertension – 12.3% and hypertension – 18.2%)^37^. In the present study, the prevalence of HBP was more common in overweight/obese and high WC (≥75^th^ percentile) participants than in those with normal BMI and normal WC. According to the analysis of literature, overweight, obese^38^ and abdominal obese^39^ children and adolescents are more likely to have cardiometabolic risk factors. A large body of evidence suggests that there are significant associations between overweight, obesity, and HBP among children and adolescents^40–43^. WC ≥ 75^th^ percentile was also found to be significantly associated with an increased risk of HBP in children and adolescents^44^. In addition, a study of subjects aged 10–14 years in Cyprus indicated that the participants with WC > 75^th^ percentile had significantly higher odds ratios of having HBP, high levels of low-density lipoprotein cholesterol and triglycerides, compared to those with WC ≤ 75^th^ percentile^45^. Obesity is associated with adipose tissue dysfunction, which leads to the activation of the renin-angiotensin-aldosterone and sympathetic systems, chronic vascular inflammation, oxidative stress, and inflammation, leading to hypertension^46^. Furthermore, in multivariate analyses, when we adjusted for risk factors such as BMI and WC, the associations between the SNP rs7129220 and HBP became stronger.

Adrenomedullin is a 52-amino acid peptide synthesized and secreted by various cells, especially vascular endothelial and smooth muscle cells^24^. *ADM* encodes a 185-amino acid preprohormone, pro-ADM, which after posttranslational modification produces a stable form of *ADM*, easily measurable in blood samples^22^. Available data show that *ADM* could have a promising and interesting role as a diagnostic and prognostic biomarker in future^22,32,47^.

Plasma ADM level is increasing in the presence of various cardiovascular diseases^22^. Inflammatory cytokines and circulating hormones can strongly increase *ADM* production^24,48^. Released from vascular wall, *ADM* acts as autocrine, paracrine, and endocrine modulator through calcitonin receptor-like receptor (CRLR) and receptor activity-modifying protein (RAMP), and participates in blood pressure homeostasis^24,49^. It has been reported that *ADM* increases cAMP (cyclic adenosine monophosphate) levels and activates protein kinase A in vascular endothelial and smooth muscle cells^29^. In addition, *ADM* increases the synthesis of NO and cyclic guanosine monophosphate (cGMP) by binding to the receptors on the endothelium. *ADM* induces NO production by activating phosphatidylinositol 3-kinase and Akt phosphorylation via Ca^2+^/calmodulin-dependent pathway, which induces endothelium-dependent vasodilation and affects the vascular tone^28,50^.

Studies have shown that in humans, plasma levels of *ADM* are higher in patients with various cardiovascular diseases, including hypertension, and correlate with disease severity^22,26,51^. Intravenous infusion of *ADM* elicits dose-dependent blood pressure reduction leading to a decrease in total peripheral resistance^28,52^. These findings suggest that there is a possible direct and protective relationship between *ADM* release and an increased blood pressure. However, there are data showing that plasma *ADM* levels remain high in patients despite efficient antihypertensive therapy^53^. In humans, the *ADM* gene is located at chromosome 11, and genetic variations in the *ADM* gene may determine blood pressure levels and the risk for hypertension; however data from various studies are inconsistent^51,54–56^. In a Japanese study, a microsatellite marker of cytosine-adenine repeats was associated with hypertension, but in another Japanese population, this marker and other polymorphisms of the *ADM* gene (rs4399321 and rs7944706) had no association with hypertension^54^. In a family-based Chinese population study, rs3814700 in the *ADM* gene was linked with SBP and PP^55^. In a large Chinese population study, no significant association between three SNP (rs4399321, rs4910118, and rs7944706) of the *ADM* gene and hypertension were observed, but SNP rs4399321 was found to be associated with BP levels among controls^56^.

In our study, we found that the polymorphism rs7129220 was associated with higher mean DBP and MAP. The minor allele carriers had higher odds of having HBP in different inherence models. Significant associations were found in additive models for both sexes separately: among boys – aOR = 2.11; P = 0.014 (for hypertension), while among girls – aOR = 2.79; P = 0.021 (for prehypertension). Thus, our results support the hypothesis that rs7129220 or the nearest locus could play an important role in the pathogenesis of hypertension, however further studies are needed to clarify the actual mechanism of this polymorphism in blood pressure regulation.

In conclusion, we detected that the risk allele of the SNP was associated with higher odds of having high blood pressure in Lithuanian adolescents aged 12–15 years. These results are consistent with the hypothesis that *ADM* might play a protective role in hypertension. However further functional investigation and replications in other populations are needed to confirm the association of *ADM* with blood pressure.

## Limitations

There are some limitations in our study. The major limitation is that plasma concentration of *ADM* was not measured in relation to studied genetic variant. Therefore we can’t evaluate the effect of rs7129220 on the plasma level of *ADM* and to provide more detailed insights on the mechanisms leading to the increased blood pressure by this genetic variant.

The absence of detailed information on the family history of cardiovascular diseases or the socioeconomic status also limited our research as well as lack of information on the pubertal status of children and adolescents. In our study, BP was measured using an automatic oscillometric device, which has been clinically validated^57^, although, according to the Fourth Report^58^, BP measurements should be performed using auscultation. This is a cross-sectional study design, and thus we cannot determine a cause-effect relationship. In addition, various biases (for example, selection, information, and confounding) can influence the results in an observational study^59^.

## Methods

### Study design and population

This is a cross-sectional study of schoolchildren who had participated in the baseline survey “Prevalence and Risk Factors of HBP in 12–15-Year-Old Lithuanian Children and Adolescents (Study 1, 2010–2012)” in Kaunas city and Kaunas district, which are the second largest city and district in Lithuania. A written informed consent for the participation in the study was obtained from each participant’s parent or guardian. The study was approved by Kaunas Regional Biomedical Research Ethics Committee at the Lithuanian University of Health Sciences (protocol No. BE-2-69). All methods were performed in accordance with relevant guidelines and regulations. More details regarding the above–mentioned survey that was based on a two-stage cluster sampling design are given elsewhere^19^. The baseline study enrolled 7,638 children and adolescents aged 12–15 years who at the time of the examination (from November 2010 to April 2012) attended gymnasiums or secondary schools of all classes (grades 6, 7, 8, and 9) from all the invited schools that participated in the research project. The current study analyses data of a randomly selected sample of 675 adolescents (313 boys and 333 girls) aged 12–15 years who participated in the baseline study and underwent BP and anthropometric measurements and genetic testing of the saliva. The adolescents who had congenital heart defects, kidney diseases, or endocrine diseases were excluded from the study. Data on clinically verified health disorders were collected from the subjects’ medical records. The sample size was determined using the formula n = z^2^pq/d^2^, where the estimated prevalence rate of elevated BP among schoolchildren was 35%^31^, the z-value of the 95% confidence level was 1.96, and 5% was the degree of precision. The minimum sample size was calculated to be 350 subjects.

### Measurements

The methods of the performed BP and anthropometric measurements were presented in our previous article^19^.

#### Blood pressure measurement

In the morning (8:30 am to 11:30 am), after the participants had sat still for ten minutes, BP measurements were performed with an automatic BP monitor (OMRON M6; OMRON HEALTHCARE CO., LTD, Kyoto, Japan) using a cuff of the appropriate size. BP was measured three times with a 5-minute rest interval between the measurements, with the subject being in a sitting position. The mean of three BP measurements was calculated and used in the analysis. BP was measured by a physician who was not wearing a white coat. The respondents with estimated HBP (≥90^th^ percentile) at the first screening underwent the second BP assessment within a two-three week period.

Classifications of BP levels were defined according to “The Fourth Report on the Diagnosis, Evaluation, and Treatment of High Blood Pressure in Children and Adolescents”. Normal BP was defined as SBP and DBP less than the 90^th^ percentile, prehypertension was defined as an average SBP or DBP levels between ≥90^th^ percentile and <95^th^ percentile; and hypertension was defined as an average SBP or DBP ≥95^th^ percentile for sex, age, and height^58^. The mean arterial pressure (MAP) was calculated according to the traditional formula: MAP = (SBP + (2 × DBP))/3^60^, and the pulse pressure (PP) was calculated as follows: PP = SBP-DBP.

#### Anthropometric measurement

All subjects underwent anthropometric measurements while wearing only light clothes and barefoot. Height and body weight of the participants were measured to the nearest 0.1 cm and 0.1 kg, respectively, by using a portable stadiometer and a balance beam scale. The body mass index (BMI) was calculated as weight in kilograms divided by the square of height in meters. According the cut off points of BMI by age and sex for adolescents recommended by the IOTF, the participants were grouped into three categories: normal-weight, overweight, and obese^61^.

Waist circumference (WC) was measured to the nearest 0.5 cm by using a flexible measuring tape (SECA) in the standing position at a level midway between the lower rib margin and the iliac crest. The subjects were classified into the categories: below the 75^th^ percentile (normal waist value), and ≥75^th^ percentile (high waist value), according to the cut-off points of the WC percentiles published by the NHANES III^62^.

### Genetic analysis

For DNA extraction, saliva samples were collected from each individual during their health examination. DNA was extracted using a commercial DNA isolation kit – the “Genomic DNA Purification Kit” (“Thermo Fisher Scientific”, Lithuania) according to the manufacturer’s instructions.

Single-nucleotide polymorphism (SNP) of the *ADM* gene (rs7129220) was evaluated using real-time PCR with TaqMan allelic discrimination Assay-By-Design genotyping assay C__30872739_10, according to the manufacturer’s instructions (Applied Biosystems, Foster City, CA, USA). Allele-specific fluorescence was analysed on the ABI 7900HT Sequence Detection System with SDS v 2.1 (Applied Biosystems, Foster City, CA, USA).

### Statistical analysis

The normality of the distribution of continuous variables was assessed by the Kolmogorov-Smirnov test. Continuous variables were presented as mean values ± standard deviation (SD). Comparisons between the groups were performed by applying the chi-squared (χ^2^) test, Student’s t-test, and ANOVA. Categorical variables were expressed as numbers and percentages. The z test was used to compare differences between the groups. The χ^2^ test was used for the assessment of the Hardy-Weinberg equilibrium (HWE) for the distribution of genotypes.

Univariate and multivariate logistic regression analyses were conducted for both sexes separately to evaluate the associations between *ADM* gene polymorphisms and the odds of prehypertension and hypertension. Crude odds ratios (OR) and adjusted odds ratios (aOR) along with 95% confidence intervals (CI) were calculated. Logistic regression analyses were used to test for the associations of the *ADM* gene polymorphism with HBP under four inheritance models: co-dominant (wild-type homozygous vs. heterozygous; wild-type homozygous vs. minor allele homozygous), dominant (wild-type homozygous vs. heterozygous + minor allele homozygous), overdominant (wild-type homozygous + minor allele homozygous vs. heterozygous) and additive models. In multivariate analysis, ORs were adjusted for BMI and WC. The best fitting model had the lowest AIC value.

Statistical analysis was performed using the statistical software package SPSS version 20 for Windows. *P* values < 0.05 were considered to be statistically significant.

## Data Availability

According to the Statute of the Lithuanian University of Health Sciences, the authors cannot share the data underlying this study. For inquires on the data, researchers should first contact the owner of the database, the Lithuanian University of Health Sciences.
